# Absolute serum neurofilament light chain levels and its early kinetics predict brain injury after out-of-hospital cardiac arrest

**DOI:** 10.1007/s00415-021-10722-3

**Published:** 2021-07-30

**Authors:** Christoph Adler, Oezguer A. Onur, Simon Braumann, Hannes Gramespacher, Stefan Bittner, Steffen Falk, Gereon R. Fink, Stephan Baldus, Clemens Warnke

**Affiliations:** 1 Department of Internal Medicine III, Division of Cardiology, Pneumology, Angiology and Intensive Care, University of Cologne, 50937 Cologne, Germany; 2 Department of Neurology, Faculty of Medicine and University Hospital Cologne, University of Cologne, 50937 Cologne, Germany; 3 Cognitive Neuroscience, Institute of Neuroscience and Medicine (INM‐3), Research Centre Jülich, Jülich, Germany; 4 Department of Neurology, University Medical Center Mainz, Langenbeckstr. 1, 55131 Mainz, Germany; 5 Fire Department City of Cologne, Institute for Security Science and Rescue Technology, Cologne, Germany

**Keywords:** NFL, Cerebral hypoxia, Brain hypoxia, Hypoxic–ischemic brain injury

## Abstract

**Objectives:**

To test if the early kinetics of neurofilament light (NFL) in blood adds to the absolute values of NFL in the prediction of outcome, and to evaluate if NFL can discriminate individuals with severe hypoxic–ischemic brain injury (sHIBI) from those with other causes of poor outcome after out-of-hospital cardiac arrest (OHCA).

**Design and setting:**

Monocentric retrospective study involving individuals following non-traumatic OHCA between April 2014 and April 2016. NFL concentrations were determined on a SiMoA HD-1 device using NF-Light Advantage Kits.

**Participants:**

Of 73 patients screened, 53 had serum samples available for NFL measurement at three timepoints (after 3, 24, and 48 h of admission). Of these 53 individuals, 43.4% had poor neurologic outcome at discharge as assessed by Glasgow–Pittsburgh cerebral performance categories, and, according to a current prognostication algorithm, poor outcome due to sHIBI in 20.7%.

**Main outcome measure:**

Blood NFL and its early kinetics for prognostication of outcome and prediction of sHIBI after OHCA.

**Results:**

An absolute NFL > 508.6 pg/ml 48 h after admission, or a change in NFL > 494 pg/ml compared with an early baseline value predicted outcome, and discriminated severe sHIBI from other causes of unfavorable outcome after OHCA with high sensitivity (100%, 95%CI 70.0–100%) and specificity (91.7%, 95%CI 62.5–100%).

**Conclusions:**

Not only absolute values of NFL, but also early changes in NFL predict the outcome following OHCA, and may differentiate sHIBI from other causes of poor outcome after OHCA with high sensitivity and specificity. Our study adds to published data, overall corroborating that NFL measured in blood should be implemented in prognostication algorithms used in clinical routine.

## Introduction

Severe hypoxic-ischemic brain injury (sHIBI) is the major cause of death in patients successfully resuscitated following out of hospital cardiac arrest (OHCA) [1]. However, most of these deaths are not directly caused by sHIBI but rather result from withdrawal of life support (WLS) following unfavorable neuroprognostication [2, 3]. The 2021 joint guidelines of the European Resuscitation Council (ERC) and the European Society of Intensive Care Medicine recommend neuroprognostication no earlier than 72 h after return of spontaneous circulation (ROSC) and in comatose patients with a motor response ≤ 3 of the Glasgow Coma Scale (abnormal flexion, extension, or nil) [4]. The use of sedatives and targeted temperature management (TTM) is among the reasons for this suggestion. Neuroprognostication following OHCA is paramount not only to guide treating physicians and families in deciding whether or not to continue life support, but also to avoid the overuse of limited intensive care resources, even more so in light of a global pandemic.

Neurofilament proteins are part of the neuronal cytoskeleton and are released into the CSF and subsequently in the peripheral blood upon neuroaxonal damage [5]. Technical developments have introduced highly sensitive single-molecule assays (SiMoA) for reliable, easily accessible measurements of neurofilament light chain levels in blood samples (NFL). NFL is currently emerging as a diagnostic blood-marker for neuronal injury in neurodegenerative, inflammatory, and ischemic diseases of the central nervous system (CNS) [5–7]. Data on the value of NFL for prognostication following OHCA is evolving: in a biobanking study involving 29 participating sites, NFL levels in blood were shown to predict the outcome in 782 unconscious patients with OHCA of presumed cardiac origin, measured 24 to 72 h following hospital admission. NFL levels above 641.4 pg/ml at 24 h after cardiac arrest had a 99% specificity for poor outcome [8], confirming data of an earlier ELISA-based smaller cohort study [9]. These findings have independently been confirmed in recent studies: in a post-hoc analysis of another study involving 120 patients and assessment in blood taken at 48 h of admission, median NFL concentrations in plasma were found higher in patients with poor outcome compared to those with good outcome (2343 vs. 19 pg/ml), with the outcome assessed at month 6 of admission [10]. In a single-center study including 164 OHCA patients, NFL was measured already within 24 h of admission, and the median NFL values were higher in patients with poor outcome compared to those with good outcome (116 vs. 27 pg/mL) [11]. The three key studies mentioned allow the conclusion that NFL should be further evaluated for the purpose of neuroprognostication of OHCA patients in clinical routine.

Our study tested the value of NFL levels in blood samples of a monocentric cohort of OHCA patients, with a baseline sample taken within the first 3 h of admission, and then after 24 and 48 h. Thus, as the published studies lack an early baseline sampling, we aimed to test if an early increase (delta NFL) adds to the absolute values of NFL in the prediction of outcome, and to evaluate if NFL and its early kinetics can discriminate cases with sHIBI from other causes of poor outcome after OHCA.

## Methods

### Study design, setting, and patients

This monocentric retrospective study investigated NFL levels in serum of non-traumatic OHCA patients admitted to the Department of Cardiology of the University Hospital of Cologne between April 2014 and April 2016 [12].

A total of 73 individuals were screened, and patients were eligible if blood samples were available for NFL measurement at three timepoints: at admission to ICU within the first 3 h (day 0), 24 (day 1), and 48 h (day 2) after determination of OHCA. These samples were originally obtained for quantification of neuron-specific enolase (NSE) following a routine clinical protocol. Cardiac arrest was defined as apnea or agonal respiration in a comatose patient without a palpable pulse. No-flow time was only quantified in witnessed OHCA patients. ROSC was defined as a palpable pulse for at least 20 s [13, 14]. Time to ROSC was calculated from the time of determination of collapse to ROSC. According to current recommendations and the institutional clinical routine protocol, all patients received standard intensive care treatment, including TTM (32–34 °C) for 24 h.

### Clinical variables and serum neurofilament light chain determination

Neurologic outcome was assessed at the time of hospital discharge according to the Glasgow–Pittsburgh cerebral performance categories (CPC) ranging from CPC1–5 [13, 15].

The prognostication strategy algorithm proposed by the European Resuscitation Council and the European Society of Intensive Care Medicine [4] was used to identify patients with sHIBI among individuals with poor outcome (CPC3–5), independently assessed by two of the authors (HG, CW).

Serum was collected into tubes containing a clotting activator gel (S-Monovette 4 ml Z-Gel, Sarstedt, Nürnberg, Germany), allowed to clot for 30 min and centrifuged at room temperature at 2500 × g for 10 min. Spare serum samples were subsequently transferred into a 2 ml screw top polypropylene tube (Fisher Scientific, Schwerte, Germany) and stored at − 80 °C until further analysis. The NFL concentration was determined at the University Medical Center Mainz, Department of Neurology, in a blinded fashion without clinical data using a SiMoA HD-1 device (Quanterix, USA) and NF-Light Advantage Kits (Quanterix) of a single lot according to the manufacturer’s instructions. Briefly, resorufin-β-D-galactopyranoside (RGP) was incubated at 33 °C for 60 min, while both the samples and the remaining kit components were allowed to come to room temperature. The samples were then vortexed and spun for 5 min at 10,000 *g* before the assay was performed. Finally, all samples were measured in duplicates, and the coefficient of variation (CV, as a percentage) was obtained by dividing the standard deviation by the mean value of both replicates multiplied by 100. Samples with a sample CV above 20% (or missing replicate result) were measured a second time. The mean intra-assay CV of 6.0% was determined by averaging all individual sample CVs. Besides, the same two identical low and high controls, consisting of recombinant human NFL antigen, were run in duplicates at each sample run to monitor plate-to-plate variation. The mean concentration across all runs was 3.1 pg/ml for the low control and 133.2 pg/ml for the high control with inter-assay CVs of 9.2% and 4.7%, respectively.

### Study endpoint

NFL and its early kinetics for prognostication of outcome and prediction of sHIBI after OHCA.

### Statistical analysis

Groups were dichotomized into good (CPC 1–2) and poor (CPC 3–5) outcome, following the literature [13, 15]. The discriminative ability of NFL and NSE to predict the outcome at the time of discharge was evaluated using receiver operating characteristic (ROC) to calculate cut-off levels (closest distance to the upper left corner or Youden-Index). The area under the curve (AUC) and 95%-confidence interval (95% CI) were calculated, also for categorical values, such as proportions. The Mann–Whitney test was used to compare ranks. All calculations were performed using GraphPad Prism for MacOS (GraphPad Prism version 8.4.3, La Jolla, CA, USA). A *p* value < 0.05 was considered statistically significant.

## Results

### Study cohort

**Fig. 1:**
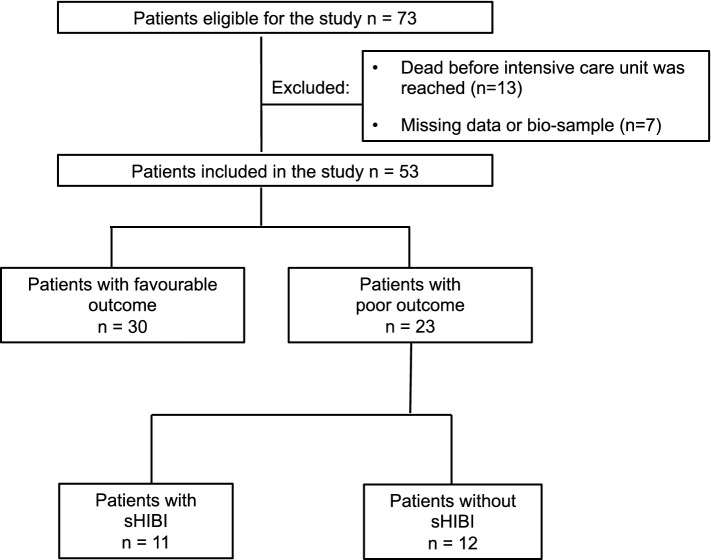
Flow of participants. sHIBI = severe hypoxic–ischemic brain injury

Fifty-three patients with NFL measurement at three timepoints (days 0, 1, and 2) were available for analysis (Fig. 1). Poor outcome (CPC3–5) was noted in 43.4% (95% CI 30.9–56.7%) of the total population. The overall cohort’s mean age was 64 years, and 83.0% (95%CI 70.6–91.0%) of the patients were male. Medical history did not differ between patients with good and poor outcomes. There was a trend of septic shock-induced OHCA, as defined by Singer and colleagues [16], occurring more often in patients with CPC3–5 (21.7%, 95%CI 9.2–42.3%) as compared to CPC1–2 (3.3%, 95%CI 0–18.1%). Witnessed cardiac arrest was more frequently reported in patients with CPC1–2 (73.3%, 95%CI 55.3–86.0%) as compared to patients with CPC3–5 (34.8%, 95% CI 18.7–55.2%). No-flow time was shorter in patients with good vs. poor outcome (Table 1**)**.

**Table 1:** Clinical characteristics in patients included in the investigation

Characteristic	Study population (*n* = 53)	Favorable outcome (CPC1–2) (*n* = 30)	Poor outcome (CPC3–5) (*n* = 23)	*p* value
Age (years ± SD)	64 ± 12	61 ± 11	67 ± 13	0.09
Female—*n* (%)	9 (17)	6 (20)	3 (13)	0.72
Medical history				
Diabetes mellitus—*n* (%)	9 (41.8)	4 (13.3)	5 (21.7)	0.48
Arterial hypertension—*n* (%)	29 (54.7)	16 (53.3)	13 (56.5)	0.78
Advanced renal disease—*n* (%)	12 (22.6)	7 (23.3)	5 (21.7)	1.00
Cardiogenic shock	47 (88.7)	29 (96.7)	18 (78.3)	0.07
Myocardial infarction—*n* (%)	32 (60.4)	18 (60.0)	14 (60.9)	1.00
Primary arrhythmia—*n* (%)	15 (28.3)	11 (36.7)	4 (17.4)	0.14
Septic shock—*n* (%)	6 (11.3)	1 (3.3)	5 (21.7)	0.07
Cardiac arrest characteristics				
Witnessed arrest—*n* (%)	30 (56.6)	22 (73.3)	8 (34.7)	< 0.01
No-flow-time (min ± SD)	4.0 ± 3.0	3.0 ± 2.2	4.8 ± 3.6	0.04
BLS provided by bystander—*n* (%)	25 (47.2)	15 (50.0)	10 (43.5)	0.78
Shockable rhythm—*n* (%)	45 (84.9)	28 (93.3)	17 (73.9)	0.06
Number of shocks ± SD	3.0 ± 4.6	3.0 ± 2.9	3.5 ± 2.8	0.58
Time to ROSC (min ± SD)	20.3 ± 16.4	15.5 ± 10.9	26.4 ± 20.1	0.03
Outcome				
Length of ICU stay (days ± SD)	17.6 ± 11.4	18.4 ± 8.1	16.4 ± 14.8	0.56
Ventilation time (days ± SD)	11.6 ± 11.2	8.6 ± 7.1	15.5 ± 14.3	0.04

*CPC* cerebral performance category, *BLS* basic life support, *ROSC* return of spontaneous circulation, *ICU* intensive care unit, *SD* standard deviation

### Correlation of NFL and NSE levels with outcome

**Fig. 2:**
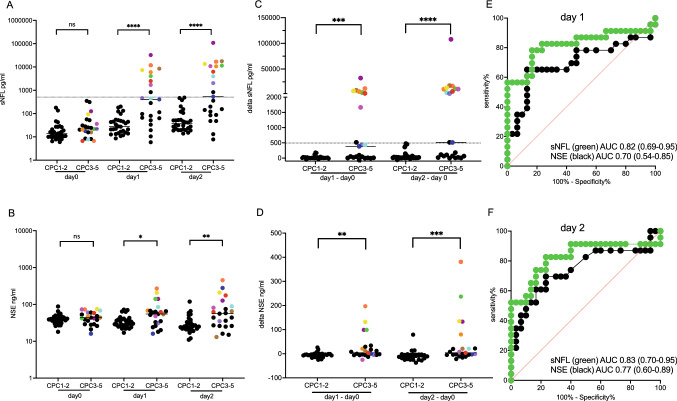
Comparison of NFL and NSE values to predict outcome after OHCA. Absolute values of NFL (**A**) and NSE (**B**) and changes to NFL (**C**) and NSE (**D**) values are shown for patients stratified based on the cerebral performance category (CPC) in good and poor outcome. Plain line: median. Individual patients with severe hypoxic–ischemic brain injury (sHIBI), as classified in Table 2, are color-coded to allow identification of single individuals. The sensitivity and specificity of absolute NFL and NSE values at day 1 (E) and day 2 (F) are compared by receiver operating characteristic (ROC) analysis. Dotted line: cut-off points for absolute NFL (508.6 pg/ml) or change in NFL (> 494 pg/ml)

Both absolute NFL and NSE levels and its kinetics were associated with poor outcomes (CPC3–5) at days 1 and 2 after CPR, but not within the first 3 h (day 0) of admission (Fig. 2A–D). ROC analysis demonstrated the superiority of NFL compared to NSE to predict poor outcome both at days 1 and 2 (Fig. 2E–F).

NFL levels above > 241.7 pg/ml resulted in a 100% specificity (95% CI 88.6–100.0%) and 56.5% sensitivity (95% CI 36.8–74.4%) to predict CPC 3–5 at day 1, and NFL levels above > 508.6 pg/ml resulted in a 100% specificity (95% CI 88.6–100.0%) and 52.2% sensitivity (95% CI 33.0–70.8%) to predict CPC 3–5 at day 2.

### NFL in patients classified as severe hypoxic–ischemic brain injury according to current prognostication algorithms

Among the patients with CPC3–5, 12 of 23 (52%) individuals had an absolute NFL > 508.6 pg/ml at day 2, and a change in NFL > 494 pg/ml comparing the value at day 2 and day 0. Eleven of these 12 patients fulfilled the criteria for “*poor outcome likely”* (termed severe hypoxic–ischemic brain injury (sHIBI), Table 2) according to the prognostication strategy algorithm proposed by the European Resuscitation Council and the European Society of Intensive Care Medicine [4]. Thus, NFL was elevated in 100% of the patients with predicted sHIBI. In other words, high or increasing NFL values had a sensitivity of 100% (95%CI 70.0–100%) and a specificity of 91.7% (95%CI 62.5–100%) for sHIBI in our cohort.

**Table 2:**
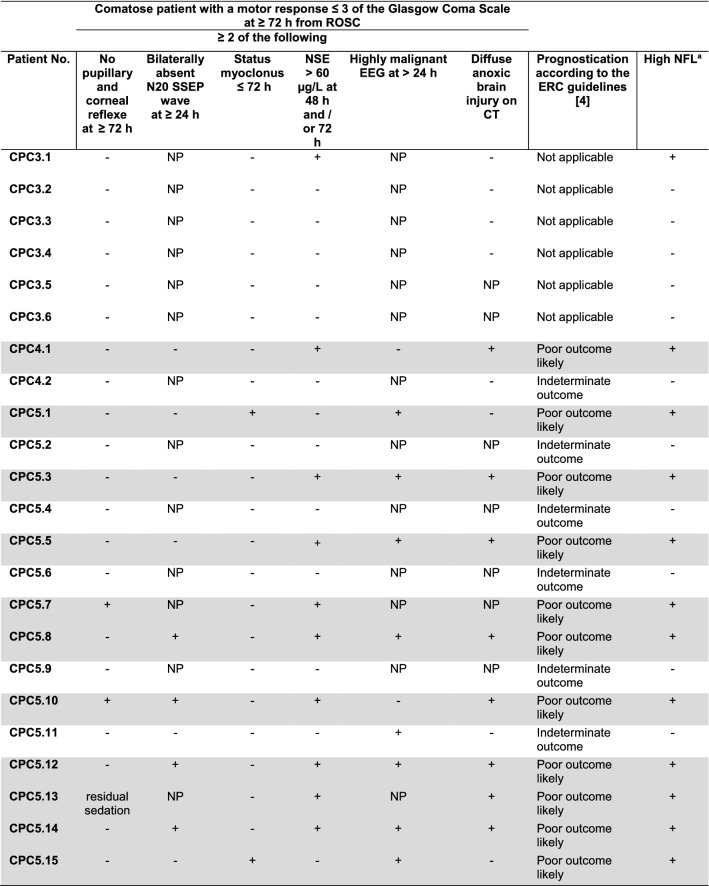
Table showing clinical features of the CPC3–5 patients and, if applicable, the prognostic classification according to the prognostication strategy algorithm proposed by the European Resuscitation Council and European Society of Intensive Care Medicine Guidelines for Post-resuscitation Care 2021

Grey fields mark the patients classified with “poor outcome likely”, considered as patients with severe hypoxic ischemic brain injury (sHIBI)

*CPC *cerebral performance categories, *SSEP* somatosensory evoked potentials, *ROSC* return of spontaneous circulation; *NSE* serum neuron-specific enolase, *NFL* serum neurofilament light chain, *EEG* electroencephalography, *CT* computed tomography, *NP* not performed, +  criterion fulfilled, —  criterion not fulfilled

^a^Absolute NFL > 508.6 pg/ml after 48 h, or a change in NFL > 494 pg/ml compared with baseline taken within 3 h of admission

## Discussion

In this study, we could confirm that NFL is a highly specific marker for poor outcome (CPC3–5) following OHCA, superior to NSE currently used in clinical practice. The cut-point at 641.4 pg/ml in the largest study published and measured at 48 h of admission was associated with CPC 3–5 in all patients of this smaller independent cohort tested [8]. As a novel observation, an increase in NFL of > 494 pg/ml comparing values at baseline (within the first 3 h of admission) and after 48 h also predicted poor outcome in our cohort. Low NFL baseline values found early after OHCA in all individuals, and the consecutive increase in patients with unfavorable outcome strongly support the validity of NFL for prognostication and corroborates the conclusion that NFL should be considered as a standard laboratory measure in the evaluation of OHCA patients [8–10]. Our finding is of importance for the design of independent larger prospective studies in which an early baseline sampling should be obtained.

As shown most clearly in Fig. 2C for the early delta NFL in patients with CPC3–5, the patients split into those with only marginal or no NFL increase, and a proportion of around 50% of patients with an increase in NFL far beyond 494 pg/ml, thereby separating two distinct populations: a population that could be classified as individuals with “poor neurological outcome likely” according to the prognostication strategy algorithm proposed by the European Resuscitation Council and the European Society of Intensive Care Medicine [4] with very high NFL values classified as sHIBI; and a population with comparably low NFL values considered as patients without sHIBI, and a poor outcome due to other causes of limited patient prognosis, such as severe multi-organ dysfunction syndrome not primarily involving the brain.

Both NSE and NFL are abundantly found in neurons and their release into the blood stream indicates neuronal damage. NSE is the most widely used and best investigated biomarker for neuroprognostication in cardiac arrest patients [17–19]. However, release of NSE into the peripheral blood is not specific for neuronal damage. NSE has not only been found in neurons but also in neuronendocrine tumors, paraneuronal tissue and even red blood cells and platelets [20–22]. Therefore, concurrent neuroendocrine tumors or massive hemolysis are among potential confounders for NSE levels, possibly explaining a lower specificity of NSE compared to NFL for neuroprognostication found in our study, and confirming the published literature. In contrast, NFL is part of neuronal cytoskeleton, only expressed in neurons and, therefore, is a highly specific marker of neuronal damage [5].

Futile medical care has many definitions [23] but typically depends on the probability of reaching goals of treatment [24]. sHIBI following OHCA is a typical scenario for medical futility. A European-wide survey revealed that most people would not like to be kept alive in case of a permanent vegetative or minimally conscious state [25]. Accordingly, Dragancea and colleagues demonstrated withdrawal of life support to be the most common cause of death in patients with OHCA that reach the ICU [2].

In times of resource-constrained healthcare systems, accurate and rapid prognostication of outcome in intensive care patients is warranted. In patients successfully resuscitated following OHCA, absolute NFL, and early changes of NFL measured in blood—in combination with standard prognostication criteria [2, 3]—has the potential to ease decision-making for medical personal and family, reduce economic impact and preserve urgently needed ICU capacities, in particular in the times of a global pandemic.

Our study has several limitations. First, it is a retrospective monocentric cohort study, with limitations inherent to study design. This resulted, e.g., in a predominance of the male gender, and a retrospective classification of patients as sHIBI based on chart review according to published algorithms. Despite the independent evaluation of clinical parameters by two of the investigators, not all further investigations required were performed in all individuals to predict outcome following OHCA, reducing the likelihood to classify individuals as sHIBI. This may have resulted in an overestimation of the predictive value of NFL for sHIBI in our study, making prospective validation mandatory. Second, absolute values of NFL measured by SiMoA published in recent years are not directly comparable due to a lack of standardization of NFL assays between different laboratories. Therefore, although our study resulted in cut-off values at 48 h to predict outcome following OHCA comparable to the largest published applying a similar technique [8], the assay validation steps were not identical, and additional external validation is needed before the use of absolute cut-off values for prognostication in clinical practice. The same applies to the establishment of the measurement of early NFL dynamics for prognostication.

## Conclusions

Our study investigating NFL levels in patients with non-traumatic OHCA corroborates previous studies showing that absolute NFL values should be considered as a standard laboratory measure in prognostication of OHCA patients. As a novel finding, we also studied NFL in blood samples early after admission allowing to assess the early NFL dynamics. An early increase in NFL also predicted poor outcome with high specificity, and may early help to identifying individuals with severe brain hypoxia. Thus, NFL measurements in blood should become broadly available to be used in the clinical routine on standard laboratory instruments to enable prospective validation, and its use for prognostication in critical care medicine.

## Data Availability

Anonymized data will be shared by request from any qualified investigator.
